# Mediation of Self-Compassion on Pathways from Stress and Anxiety to Depression among Portuguese Higher Education Students

**DOI:** 10.3390/healthcare11182494

**Published:** 2023-09-08

**Authors:** Carla Serrão, Andreia Valquaresma, Ana Rita Rodrigues, Ivone Duarte

**Affiliations:** 1 Escola Superior de Educação, Instituto Politécnico do Porto, Rua Dr. Roberto Frias, 4200-465 Porto, Portugal; 2 Center for Research and Innovation in Education, Polytechnic of Porto, 4200-465 Porto, Portugal; 3 Department of Social and Behavioural Sciences, University of Maia, 4475-690 Maia, Portugal; andreia.valquaresma@gmail.com (A.V.); arrodrigues@ismai.pt (A.R.R.); 4 Center for Vocational Development and Lifelong Learning, University of Porto, 4299-002 Porto, Portugal; 5 Center for Psychology, University of Porto, 4200-135 Porto, Portugal; 6 Faculty of Medicine, MEDCIDS-Department of Community Medicine, Information and Decision in Health, University of Porto, 4200-450 Porto, Portugal; 7 Center for Health Technology and Services Research (CINTESIS), Faculty of Medicine, University of Porto, 4200-319 Porto, Portugal

**Keywords:** depression, anxiety, stress, self-compassion, higher education, Portugal, cross-sectional study

## Abstract

Higher education is a context that requires students to develop academic, social and institutional tasks. As a result of this complex and multidimensional process, students tend to experience greater stress, anxiety and depression, making it crucial for students to mobilize a set of essential personal, social and instrumental resources, for a more positive adaptation to the academic context. Self-compassion is an adaptative emotion-regulation strategy and may help students to better adjust to academic issues. The purpose of this study was to investigate the role of self-compassion as a mediator in the relationship between anxiety and depression, as well as stress and depression. Methods: A cross-sectional study was conducted using an online questionnaire distributed through social media. Stress and anxiety were found to be positively related to depression scores and negatively related to self-compassion. A bootstrapped mediation model confirmed the existence of a significant positive partial mediation effect exerted by self-compassion on the relationship between stress and depression (b = 0.12, 95% CI [0.05, 0.18]). The analysis also showed a significant positive partial mediation effect exerted by self-compassion in the relationship between anxiety and depression (b = 0.13, 95% CI [0.08, 0.18]). Conclusions: Self-compassion might partially mediate the relationship between stress and depression and between anxiety and depression. Findings underscore self-compassion as a potentially protective factor against negative psychological symptoms.

## 1. Introduction

Higher education is a context that requires students to develop academic, social and institutional tasks [1,2]. In addition to these challenges, most college students find themselves in a transitional period between adolescence and adulthood: emerging adulthood [3]. This stage is marked by social, physical and psychological changes [3]. As a result of this complex and multidimensional process, students tend to experience greater stress, anxiety, and depression (e.g., [1,4,5,6]). Depression, anxiety and stress are important indicators for mental health [1,5] which, if untreated, can have detrimental effects on students’ development, academic performance, subjective and psychological well-being, and social relationships [1]. According to Lovibond and Lovibond’s Tripartite Model [7], depression is characterized primarily by a loss of self-esteem and incentive and is associated with a low perceived probability of achieving meaningful life goals for the individual as a person. Anxiety underlines the link between relatively persistent anxiety states and acute anxiety responses. Stress is a state of constant excitement and tension with a low threshold for excitement or depression. Stress includes nervousness, irritability, and a tendency to overreact to stressful events.

These disorders are recognized as a significant and expanding public health issue [8]. Syed et al. [8], for example, found that more than half of 267 undergraduate students experienced depression, anxiety, and stress. A recent meta-analysis of the prevalence of depression, anxiety, and stress in university students [5] reported a high prevalence of these emotional disorders, particularly among medical students.

If before the pandemic, the student’s mental health was a global concern, the pandemic context introduced several challenges that affected students’ academic lives, social connections and overall well-being. For example, in Jordan, Hamaideh et al. [4] explored the prevalence of depression, anxiety, and stress among 1380 university students during the COVID-19 outbreak’s “home-quarantine”. The results showed that students are experiencing moderate to severe levels of psychological distress and stress levels that are higher than those seen in the pre-COVID period.

Psychological distress and stress are the most prevalent mental disorders, with Portugal ranking fourth in Europe in terms of prevalence [9]. However, little is known about the mental health of Portuguese higher education students and no baseline data have been available to make accurate comparisons. According to the Portuguese Directorate-General for Health (DGS) report on the National Mental Health Program, 21% of the population had an anxiety disorder and 17% experienced depression [10]. 

Although students have to deal with multiple academic stressors that can trigger psychological issues, not all experience these problems. Self-compassion can be a protective variable against mental illness [11,12,13]. 

### Self-Compassion: Concept and Correlates

Self-compassion is a concept that stems from Buddhism and it was first introduced by Kristin Neff [11]. Compassion for oneself is referred to as self-compassion. Self-compassion, according to Neff [11], consists of three interconnected dimensions, each of which has two parts: the presence of one construct and the absence of another. This multidimensional construct includes (1) self-kindness: being kind to oneself rather than being critical of oneself; (2) common humanity: seeing one’s imperfections as part of the higher human condition rather than feeling isolated; and (3) mindfulness: holding one’s painful thoughts and feelings in mindful awareness rather than avoiding or overidentifying with them [“one’s sense of self becomes so immersed in one’s subjective emotional reactions that it becomes difficult to distance oneself from the situation and adopt a more objective perspective” [11] (p. 224)]. Taken together, these different dimensions represent a self-compassionate state of mind [12]. According to Rodrigues and Serrão [14], compassion is not about an intellectual, surface-level understanding that does not bring about transformation. Instead, it is a deep emotional understanding of suffering as a human experience, allowing the emergence of warm feelings (love, kindness).

Individuals who are “self-compassionate are less probable to suppress unwanted thoughts” and emotions and are more likely to recognize that their emotions are “valid and important than those who lack self-compassion” [11]. In self-compassion, the acceptance of the person one is, involves a genuine recognition that all human beings make mistakes and that wrong decisions and feelings of regret are inevitable. In this authenticity, a friendly relationship with the self is assumed [14].

In the presence of self-compassion, positive emotions are generated by welcoming negative ones [13]. Thus, it has been suggested that higher levels of self-compassion are associated with greater well-being [13], and that they are related to factors such as “positive affect, life satisfaction, optimism, happiness” and “wisdom” [15,16]. Following the compassion theory model [11], compassion activates one’s soothing emotional system, which is associated with good mental health. In this light, self-compassion is viewed as a resilience mechanism against psychopathology that is important for coping with adversity, e.g., [11]. As a result, when individuals face stressful situations or events that can lead to depression and anxiety, it is plausible to consider self-compassion as a variable that, rather than explaining how the relationship between stress, depression, and anxiety can change, can effectively explain why they are related (i.e., playing a mediating role).

In fact, growing evidence suggests that higher self-compassion scores appear to be negatively correlated with self-criticism, depression, anxiety, rumination, and thought suppression [11,13,17,18]. In this regard, MacBeth and Gumley [19] performed a meta-analysis to evaluate the strength of the association between compassion and depression, and anxiety and stress. The authors [19] reported a large effect size between compassion and psychopathology, with higher levels of compassion present in individuals with lower levels of mental health symptoms.

Although the associations between anxiety, stress, depression and self-compassion are a very debated issue in the literature, the mediation effect of self-compassion in the association between stress and depression and in the relationship between anxiety and depression are understudied topics, with scarce scientific evidence, particularly in young people and higher education students.

Thus, grounded in the hypothesis that self-compassion could mediate the relationship between stress and depression and between anxiety and depression, we designed this study aiming to explore the mediating role of self-compassion in the relationship between anxiety and depression, and between stress and depression.

## 2. Materials and Methods

### 2.1. Study Design and Participants

The data were collected cross-sectionally using a web survey distributed on social networks (e.g., Instagram, Facebook, etc.) and spread through the snowball sampling technique. The questions were answered online through the Google^®^ Forms platform. The data collection period spanned from 10 to 28 December 2021. As inclusion criteria, subjects had to be between the ages of 18 and 29 [1] and studying in a Portuguese higher education institution during the data collection period. Accordingly with the Helsinki Declaration principles, ethical procedures were considered and the study was approved by an independent Ethical Committee before data were collected (Ethics Committee of the Center for Research and Innovation in Education (InED)—Ref PAI17/CE/21 on 7 December 2021). In accordance with the General Data Protection Regulation guidelines for clinical research, all participants provided informed consent online.

From a total of 259 participants, 7 respondents were excluded because of an incomplete survey and 11 were excluded for not being higher students. The questionnaire was completed by 241 students from 21 Portuguese higher education institutions. Females outnumbered males by a wide margin (75.9%). This distribution of participants was unbalanced considering that the feminization rate in Portuguese higher education institutions is around 54% (data from 2021, PORDATA: https://www.pordata.pt/Portugal/Alunos+matriculados+no+ensino+superior+total+e+por+sexo-1048, accessed on 8 June 2023). However, we found that a sample of 74 participants was required to obtain a large effect size (f^2^ = 0.35) when we used GPower 3.1 to calculate sample size and power. Given that the study’s overall sample substantially outnumbered this value, we chose to keep the sample because it was closer to representativeness than simply excluding the male [20] and because it partially reflected the distribution of Portuguese higher education students. The participants’ mean age was 20.3 years old (SD = 1.9 years old) and the majority were single (96.7%). As for the year of studies, 29.9% said they were in their 1st year, 26.6% said they were in their 2nd year and 43.6% indicated they were in their 3rd, 4th or 5th year. Table 1 presents the socio-demographic characteristics of the participants. 

### 2.2. Measures

The data were collected via a self-report online questionnaire which included informed consent, socio-demographic data, and psychometric measures to assess depression, anxiety, stress, and self-compassion. Personal information, namely gender, age, marital status, and academic year were among the socio-demographic data collected. 

The Portuguese version of the Depression, Anxiety and Stress Scale (DASS-21, [7,21]) was used to assess depression, anxiety, and stress levels among respondents. The scale consists of 21-items divided into three subscales, each with 7 items. The response scale ranges from zero (did not apply to me at all) to three (applied to me very much or most of the time). In the present study, reliability was measured through Cronbach’s alpha. All subscales demonstrated high internal consistency, with values of 0.92, 0.90, and 0.90 for the depression, anxiety, and stress subscales, respectively. 

To assess self-compassion, the Portuguese Self-Compassion Scale (SCS, [11,22]) was used. There are 26 items on this scale and six subscales: self-kindness, self-judgment, common humanity, isolation, mindfulness, and over-identification. The self-kindness, common humanity, and mindfulness scores are added together with the reverse scores of the self-judgement, isolation, and over-identification subscales to compute the SCS total score. Higher scores indicate a higher level of self-compassion. Cronbach alpha values of 0.85 (total SCS score), 0.83 (self-kindness), 0.87 (self-criticism), 0.79 (common humanity), 0.83 (isolation), 0.77 (mindfulness), and 0.83 (over-identification) were found in the current study.

### 2.3. Statistical Analysis

To perform the data analysis, two pieces of software were used: SPSS^®^ Statistics (version 27.0, IBM, Armonk, NY, USA) and Jamovi software, version 2.3.28 (datalab.CC, Sydney, Australia). To present the descriptive statistics of the demographic and psychological variables, the mean (M), standard deviation (SD), number (N) and percentage (%) are shown. To determine between-group differences, *t*-tests and a one-way analysis of variance (ANOVA) were used. In order to analyze the associations between the SCS total score and the DASS’s subscales, Pearson correlations were calculated at a 5% level of statistical significance. The correlation coefficients were also interpreted in terms of the effect size, according to Cohen’s [23] conventions: an r of 0.10 is considered small (weak association), an r of 0.30 is considered medium (moderate association) and an r of 0.50 is considered large (strong association). 

**Figure 1:**
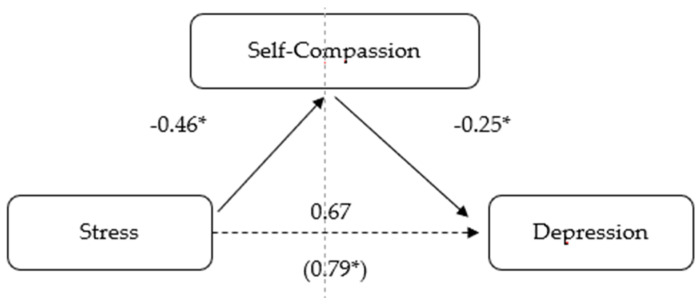
Model of stress as a predictor of depression, mediated by self-compassion. (* *p* < 0.001).

**Figure 2:**
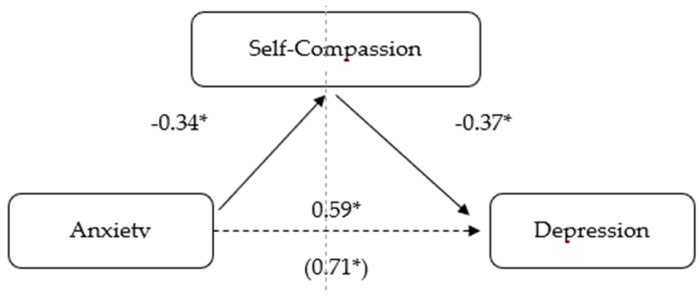
Model of anxiety as a predictor of depression, mediated by self-compassion. (* *p* < 0.001).

The possibility of a causal relationship was investigated using correlational analysis by examining the potential mediation effect of self-compassion on the relationship between the variables stress and depression, as well as anxiety and depression. To put this hypothesis to the test, we computed a mediation analysis using bootstrapping sampling with bias correction, as recommended by Preacher and Hayes [24]. The bootstrapping method has been validated in the literature and is thought to be superior to other methods of mediation analysis [25]. It estimates indirect effects through one or more mediating variables, considering bootstrap confidence intervals [24]. Thus, it includes the estimation of direct, indirect and total effects. In our case, the direct effects correspond to the effect of stress on depression and to the effect of anxiety on depression, without taking into account the possible effect of self-compassion. The indirect effects are observed in the trajectories that link stress and depression, and anxiety and depression, via self-compassion. The sum of the direct and indirect effects of stress or anxiety on depression are the total effects (see Figure 1 and Figure 2). As described by Preacher and Hayes [24], mediation is demonstrated when the indirect effect is significant, and the confidence intervals do not contain zero. To calculate the mediation analyses, we installed the macro for SPSS—PROCESS, created by Hayes [21]. Following Hayes’ [25] suggestions, the present study used a 95% confidence interval and calculated 5000 bootstrap samples. The mediation model selected was the simple mediation one.

## 3. Results

### 3.1. Correlations among Depression, Anxiety, Stress and Self-Compassion

The correlations between depression, anxiety, stress, and self-compassion were all statistically significant (*p* < 0.001) and are shown in Table 2. Anxiety and stress were positively related to depression symptoms, whereas self-compassion was negatively related to depression, anxiety and stress. The correlation between the self-compassion scale (SCS), and the stress [r(239) = −0.53, *p* < 0.001] and the depression subscales [r(239) = −0.55, *p* < 0.001] of the DASS was positive and revealed a large effect. The correlations between the SCS and the anxiety [r(239) = −0.41, *p* < 0.001] subscale were positive and showed a medium effect.

### 3.2. Prevalence Rates of Depressive, Anxiety and Stress Symptoms and Sociodemographic Factors

In accordance with the DASS cutoff values [7] for the various categories of depression, 90% of the respondents scored no depression, 3.7% mild depression, 1.7% moderate depression, 1.2% severe depression, and 3.3% extremely severe depression. The different severity thresholds of the depressive symptoms were significantly associated with the course year [F(2, 238) = 6.49, *p* = 0.002, η^2^ = 0.05], but not with gender [t(85, 78) = 1.19, *p* = 0.24] or working student status [t(102.56) = 0.08, *p* = 0.93]. Bonferroni’s post hoc comparisons showed that the proportion of depression was higher among first-year students than among third-year students.

In terms of the categories of anxiety, 89.2% of the respondents reported no anxiety, 4.6% mild anxiety, 2.5% moderate anxiety, 1.2% severe anxiety, and 2.5% extremely severe anxiety. The different severity thresholds of anxiety symptoms were significantly associated with gender [t(95, 50) = 3.75, *p* < 0.001; d = 0.55] and the course year [F(2, 238) = 7.01, *p* = 0.001, η^2^ = 0.06], but not with working student status [t(106.45) = 0.32, *p* = 0.75]. Bonferroni‘s post hoc comparisons showed that the proportion of anxiety was higher among first-year students than among third-year students.

In relation to stress, 83% of the respondents revealed no stress, 6.2% mild stress, 3.3% moderate stress, 4.6% severe stress, and 2.9% extremely severe stress. The different severity thresholds of stress symptoms were significantly associated with gender [t(83,94) = 2.86, *p* < 0.005; d = 0.45] and course year [F(2, 238) = 6.17, *p* = 0.002, η^2^ = 0.05], but not with working student status [t(106.45) = 0.32, *p* = 0.75]. Bonferroni’s post hoc comparisons indicated that the proportion of stress was higher among first-year students than among third-year students.

### 3.3. The Mediating Role of Self-Compassion in the Relationship between Stress and Depression

As shown in Figure 1 and Table 3, the direct effect of the variable stress on depression, ignoring the mediator (self-compassion), was found to be positive and statistically significant (b = 0.67, SE = 0.05, *p* < 0.001). The indirect effect of stress on the mediator self-compassion was negative and statistically significant (b = −0.46, SE = 0.05, *p* < 0.001). The third step in the mediation analysis revealed that the indirect effect of the mediator controlling for the independent variable (stress) was negative and statistically significant (b = −0.25, SE = 0.06, *p* < 0.001). Finally, the mediation analysis showed that after controlling for self-compassion, stress had a statistically significant effect (b = 0.79, SE = 0.05, *p* < 0.001), indicating the presence of a positive and significant partial mediation effect exerted by self-compassion on the relationship between stress and depression (b = 0.12, 95% CI [0.05, 0.18]).

### 3.4. The Mediating Role of Self-Compassion in the Relationship between Anxiety and Depression

According to Figure 2 and Table 4, the direct effect of anxiety on depression, ignoring the mediator self-compassion, was positive and statistically significant (b = 0.59, SE = 0.05, *p* < 0.001). The indirect effect of anxiety on the mediator self-compassion was negative and statistically significant (b = −0.34, SE = 0.05, *p* < 0.001). Regarding the indirect effect of the mediator self-compassion controlling for the independent variable (anxiety), it was found to be statistically significant and negative (b = −0.37, SE = 0.06, *p* < 0.001). Finally, the mediation analysis showed that controlling for self-compassion, anxiety had a statistically significant and positive effect (b = 0.71, SE = 0.05, *p* < 0.001), which supports the existence of a positive and significant partial mediation effect exerted by self-compassion in the relationship between anxiety and depression (b = 0.13, 95% CI [0.08, 0.18]).

## 4. Discussion

This study was designed to analyze the mediating role of self-compassion in the relationship between stress and depression, and in the relationship between anxiety and depression in Portuguese higher education students.

In the current study, the prevalence of depression was 10%, anxiety was 10.8% and stress symptoms was 17%, which is lower than the prevalence reported by other studies prior [6,8] or during COVID [26,27], using the DASS in student samples. Syed et al. [8], for example, reported a prevalence of depression, anxiety and stress symptoms in a sample of undergraduate physiotherapy students of 48%, 68.5%, and 53.2%, respectively. In addition, Fuad et al. [26], reported a prevalence of depression, anxiety, and stress symptoms of 60.2%, 76.2%, and 46.9%, respectively, in a study with medical students. Our results contrast also with Silva’s study [27], with a sample of 247 Portuguese university students. The study was conducted between 2nd and 20th of July of 2020 and the results indicated that more than 40% students experienced anxiety, depression and stress symptoms. It should be highlighted that the mixed results reported in these studies may be attributable to the fact that data were gathered at various points during the pandemic, in various locations, and with various infection rates and restrictions. For example, in the present study, the data were collected a year and nine months after the onset of pandemic, and included a few days of the Christmas holiday break, factors that may constitute a relief from stressful events.

Furthermore, the findings of our study indicate a statistically significant difference in DASS symptoms based on the course year. Depression, anxiety, and stress were all higher among first-year students than among third-year students. This may be explicated by the exclusive nature of the obstacles encountered during the first year of the course, which are motivated by the need to adjust to the transfer and adapt to new routines, demands, and obligations. Previous research (e.g., [28]) indicates that students, particularly first-year higher education students, who have to move to another city, facing challenging and novel circumstances, appear to be particularly vulnerable to these types of mental disorders [29]. In line with this, many first-year college students have new friends and teachers to adjust to, as well as a new range of social and physical environments [6,30] to adapt to, so failure to cope with these transitions and their demands can increase the likelihood of developing mental illness (e.g., depression, anxiety, stress).

Moreover, the current findings revealed a statistically significant difference in anxiety and stress scores depending on gender. However, no substantial gender differences in depression were found.

Consistent with previous research, depression was found to be strongly and positively correlated to stress and anxiety in our sample (e.g., [31,32]). This finding seems to suggest that depressive symptoms were more prevalent in those who reported greater levels of stress and anxiety. Furthermore, these results show that self-compassion was negatively linked with depression and anxiety, which is in line with earlier studies [33,34,35]. In fact, self-compassion is a metacognitive activity that enables “the recognition of the related experiences of self and other, thus breaking the cycle” [36], p. 264, of over-identification or self-criticism, which reduces self-centeredness feelings of separation and isolation whilst enhancing feelings of connection [36]. According to Neff et al. [36], p.264, “when individuals feel compassion for others, they allow themselves to be touched by another’s experience of suffering”. As soon as we see the human being as a real person, who suffers, we connect with him/her and their emotional pain. Instead of ignoring their suffering, as a result of these feelings of connectedness and interconnectedness, we have an intrinsic desire to help and somehow alleviate their suffering [37]. Thus, people who have self-compassion are more likely to moderate their reactions during times of failure or difficulty, by mitigating rumination and amplifying self-reflection responses [11,12,13]. Self-compassion may, then, play a role in adaptive self-regulation by regulating negative mood states. Since it implies feelings of loving kindness, care and understanding for people who are in emotional pain (including oneself), the intention to alleviate suffering naturally arises [36]. Additionally, this recognition of human suffering involves understanding the shared human condition (every human being suffers), which reduces feelings of isolation and negative and painful emotional states [37].

Overall, the results of the mediation analyses performed support the premise that self-compassion mediates the association between stress and depression. These findings suggest that the impact of stress on depression is partially mediated by self-compassion, which means that students who are less self-compassionate are more likely to have depression symptoms when exposed to stressors events. This would indicate that improving self-compassion can reduce the symptoms of depression, both directly and indirectly, through mediating effects.

Furthermore, the mediation analysis of the present study confirmed that self-compassion played a significant mediating role in the relationship between anxiety and depression among Portuguese higher students. Higher levels of anxiety were associated with lower levels of self-compassion, which in turn, contributed to students’ higher depression levels. Anxiety is commonly exacerbated by an individual’s over-identification with their failures, shortcomings or successes. Therefore, individuals self-focus in their suffering and dramatize their situation, ignoring all else. However, the self-compassion cultivation allows the development of the opposite: individuals hold their suffering in mindful awareness without over-identifying with it, e.g., as shown in [33,34,36]. In other words, self-compassion can be trained and “requires holding suffering in balanced awareness without getting lost and over-identifying with the experience” [38], p. 25.

This study has limitations. First, it is grounded on a web-based questionnaire, distributed via email and social networks, which could have been influenced by a self-selection bias because individuals who choose to participate are not randomly selected. This could lead to a sample not fully representative of the broader population. We can hypothesize that students that were more prone to respond to the study were those experiencing lesser mental health issues. Second, the data were self-reported. As a result, possible social desirability biases cannot be ruled out. Third, because this was a cross-sectional study, the mediation results must be interpreted with caution and cannot be generalized to other populations or contexts. Fourth, despite a reasonable absolute sample size, the sample’s characteristics must represent the population for the findings to be generalizable. In this study, the sample exhibits a gender bias. There was a large number of female students compared to males, which may underrepresent male academic students (data from 2021, 46.4% males and 53.6 females; PORDATA: https://www.pordata.pt/Portugal/Alunos+matriculados+no+ensino+superior+total+e+por+sexo-1048, accessed on 8 June 2023).

## 5. Conclusions

In conclusion, when comparing our results with national or international studies, we observed a slightly lower prevalence of anxiety, depression and stress levels. Stress and anxiety were negatively related to depression, and self-compassion (as a general variable) played a partial mediating role in this relationship. Altogether, these findings emphasize the relevance of exploring and including self-compassion as a key variable when seeking to reduce individuals’ levels of stress, anxiety and depression [2].

Accordingly, our findings highlight the importance of developing intervention programs for higher education students that include self-compassion-based interventions, as well as essential and appropriate social and psychological support services aimed at treating or preventing emotional disorders and enhancing psychological well-being (e.g., [39]).

## Figures and Tables

**Table 1:** Sample socio-demographic characterization (*n* = 241).

	*n* (%)
Gender	
Female	183 (75.9)
Male	54 (22.4)
Rather not say	4 (1.7)
Civil status	
Married	8 (3.3)
Single	233 (96.7)
Academic year	
1st	72 (29.9)
2nd	64 (26.6)
3rd, 4th, or 5th	105 (43.6)
Student worker status	64 (26.6)

**Table 2:** Correlations between all SCS and DASS subscales (stress, anxiety, depression) (*n* = 241).

	DASS_Stress	DASS_Anxiety	DASS_Depression
**DASS_Anxiety**	0.80 **	--	
**DASS_Depression**	0.75 **	0.71 **	--
**SCS**	−0.53 **	−0.41 **	−0.55 **

** *p* < 0.001.

**Table 3:** Mediation analysis results (stress—self-compassion—depression).

Antecedent		Consequent						
		M (Self-Compassion)				Y (Depression)		
		Coef.	SE	*p*		Coef.	SE	*p*
X (Stress)	a	−0.46	0.05	<0.001	c’	0.67	0.05	<0.001
M (Self-Compassion)		------------	---------	---------	b	−0.25	0.06	<0.001
constant	iM	3.54	0.08	<0.001	iY	0.86	0.22	<0.001
		*R*^2^ = 0.28 F(1, 239) = 93.53, *p* < 0.001				*R*^2^ = 0.59 F (2, 238) = 172.48, *p* < 0.001		
		

**Table 4:** Mediation analysis results (anxiety—self-compassion—depression).

Antecedent		Consequent						
		M (Self-Compassion)				Y (Depression)		
		Coef.	SE	*p*		Coef.	SE	*p*
X (Anxiety)	a	−0.34	0.05	<0.001	c’	0.59	0.05	<0.001
M (Self-compassion)		------------	---------	---------	b	−0.37	0.06	<0.001
constant	iM	3.23	0.07	<0.001	iY	1.55	0.19	<0.001
		*R*^2^ = 0.17 F(1, 239) = 48.44, *p* < 0.001				*R*^2^ = 0.58 F (2, 238) = 166.27, *p* < 0.001		
		

## Data Availability

The exact data can be obtained from the corresponding author.
